# Passive immunotherapy for influenza A H5N1 virus infection with equine hyperimmune globulin F(ab')_2 _in mice

**DOI:** 10.1186/1465-9921-7-43

**Published:** 2006-03-23

**Authors:** Jiahai Lu, Zhongmin Guo, Xinghua Pan, Guoling Wang, Dingmei Zhang, Yanbin Li, Bingyan Tan, Liping Ouyang, Xinbing Yu

**Affiliations:** 1 Sun Yat-sen University, Guangzhou 510080, China; 2 Kunming General Hospital of Chengdu Military Area, Kunming, 650000, China; 3 Haerbin Veterinary research institute, Haerbin, 150000, China

## Abstract

**Background:**

Avian influenza virus H5N1 has demonstrated considerable pandemic potential. Currently, no effective vaccines for H5N1 infection are available, so passive immunotherapy may be an alternative strategy. To investigate the possible therapeutic effect of antibody against highly pathogenic H5N1 virus on a mammal host, we prepared specific equine anti-H5N1 IgGs from horses vaccinated with inactivated H5N1 virus, and then obtained the F(ab')_2 _fragments by pepsin digestion of IgGs.

**Methods:**

The horses were vaccinated with inactivated H5N1 vaccine to prepare anti-H5N1 IgGs. The F(ab')_2 _fragments were purified from anti-H5N1 hyperimmune sera by a protocol for 'enhanced pepsin digestion'. The protective effect of the F(ab')_2 _fragments against H5N1 virus infection was determined in cultured MDCK cells by cytopathic effect (CPE) assay and in a BALB/c mouse model by survival rate assay.

**Results:**

By the protocol for 'enhanced pepsin digestion', total 16 g F(ab')_2 _fragments were finally obtained from one liter equine antisera with the purity of over 90%. The H5N1-specific F(ab')_2 _fragments had a HI titer of 1:1024, and the neutralization titre of F(ab')_2 _reached 1: 2048. The *in vivo *assay showed that 100 *μ*g of the F(ab')_2 _fragments could protect BALB/c mice infected with a lethal dose of influenza H5N1 virus.

**Conclusion:**

The availability of highly purified H5N1-specific F(ab')_2 _fragments may be promising for treatment of influenza H5N1 infection. Our work has provided experimental support for the application of the therapeutic equine immunoglobulin in future large primate or human trials.

## Background

In recent years, it has become clear that human infections with highly pathogenic influenza (HPAI) H5N1 viruses are associated with severe, often fatal disease. In 1997 in Hong Kong, avian influenza A (H5N1) infected both chickens and humans. During this outbreak, 18 people were hospitalized and 6 of them died [1-3]. In February 2003, two cases of avian-like H5N1 influenza virus infection occurred among members of a Hong Kong family who had traveled to mainland China; one person recovered, the other died [4]. In 2004 and 2005, HPAI H5N1 outbreaks were reported in several Asian countries, and these outbreaks were not easily halted. Up to March 1 2006, the total number of confirmed human cases of influenza H5N1 had amounted to 174, of which 94 were fatal [5]. It cannot excluded that the additional cases were ignored in the involved countries due to a lack of clinical awareness, active surveillance, or diagnostic facilities [6].

In the early epidemic, domestic cats, captive tigers, and leopards also died from avian influenza H5N1 viruses, which indicates that H5N1 virus can cross species barriers [7,8]. More and more mammals may become involved in this epidemic. The continued circulation of the H5N1 virus in poultry increases its opportunity to adapt to humans through mutation or genetic reassortment in humans or intermediate mammalian hosts. Therefore, the ongoing H5N1 influenza epidemic in Asian bird populations poses risks to the public as well as to animal health [9]. In addition, a limited number of possible human-to-human transmissions of influenza H5N1 have been reported [10], which should serve as a prewarning of a future influenza pandemic. A human pandemic with H5N1 virus could potentially be catastrophic because of an almost complete lack of antibody-mediated immunity to the H5 surface protein in most human populations and the virulence of this viral subtype.

Although vaccines against the H5N1 virus are under development in several countries, no vaccine is ready for commercial production. The traditional inactivated vaccine production against H5N1 virus is complicated because of the requirement for high biosafety containment facilities, and the difficulty, in some cases, to obtain high virus yields in embryonated eggs due to the virus' pathogenicity [11,12]. Several other approaches have been used in an attempt to overcome these obstacles, including the use of reverse genetics techniques, generation of recombinant hemagglutinin, DNA vaccination and the use of related apathogenic H5 viruses with and without different adjuvants [13-16]. However, there is still a long way to obtain a safe and effective vaccine for preventing H5N1 virus infection in human.

Currently, two classes of drugs are available with antiviral activity against influenza viruses: the M2 inhibitors (amantadine and rimantadine), and the neuraminidase inhibitors (oseltamivir and zanamivir). Some currently circulating H5N1 strains are fully resistant to the M2 inhibitors [17,18]. For cases of human infection with H5N1, the neuraminidase inhibitors may improve prospects of survival, if administered early, but the clinical evidence is limited. Antiviral resistance to neuraminidase inhibitors has been clinically negligible so far but is likely to be detected during widespread use during a pandemic [19].

Development of H5N1-specific antibodies may be an alternative strategy for the treatment of infection and the prevention and control of future outbreaks. Previous study has shown that neutralizing Fab fragments of a hemagglutinin-specific antibody were effective in treating established influenza A virus infection in mice with severe combined immunodeficiency [20]. Ramisse *et al*. also verified that topical administration of polyvalent plasma-derived human immunoglobulin and F(ab')_2 _can protect BALB/c mice infected with a lethal dose of influenza virus [21]. Although the genus difference exists between human and mice, this strategy still deserves our attention in the treatment of a severe illness such as influenza H5N1.

The practice of administering polyclonal immunoglobulins from hyperimmune sera of animal or human origin has been used extensively in prophylactic as well as therapeutic settings, including rabies and hepatitis [22]. Excepting certain viral illnesses like measles, animal sera were used routinely due to the fact that obtaining sufficient human convalescent sera for therapeutic purpose was impractical. In the setting of viral infection, equine antiserum has been applied as an antiviral regimen to control infection by ebola [23], rabies [24,25], hepatitis B virus [26,27] and HIV [28,29]. The equine antiserum possibly results in the anaphylactoid severe acute side effects induced by contaminants including serum proteins, Fc and other fragments or aggregates [30,31]. Non-traditional antibody production methods, however can assure safety and availability of heterogenous antisera [32].

Jones and Landon reported that high yields of F(ab')_2 _fragments with high purity can be obtained from ovine antiserum by a protocol for 'enhanced pepsin digestion' [33]. To investigate the therapeutic efficacy of equine antibody to H5N1 virus, we isolated serum IgGs from horses vaccinated with inactivated H5N1 vaccine and prepared F(ab')_2 _fragments by this protocol. We report herein the protective effects of F(ab')_2 _against H5N1 virus infection in a cultured MDCK cell line and in a BALB/c mouse model.

## Materials and methods

### Virus

Influenza virus A/Chicken/Guangdong/04 (H5N1) was propagated in the allantoic cavity of embryonated hen's eggs. The titer of infectious virus was determined by limiting dilution in microcultures of Madin-Darby canine kidney (MDCK) cells and was expressed as the 50% tissue culture infectious dose (TCID_50_). Infectious stocks typically contained ~10^8.5 ^TCID_50_s/ml. Aliquots were stored frozen (-70°C) and used once for infection of mice or determination of antibody-mediated virus neutralization activity. All operations with H5N1 virus were performed in a bio-safety level 3 (BSL-3) laboratory.

### Antiserum

An inactivated influenza H5N1 vaccine strain isolated in 2003, provided by the South China Agricultural University, was performed for four times immunization of health horses according to the operating procedures recommended by State Food and Drug Administration (SFDA) (not shown). The hyperimmune sera from immunized horses were collected and stored at -80°C until used.

### Preparation of equine F(ab')_2 _fragments

To prepare the F(ab')_2 _fragments, 500 ml hyperimmune sera from immunized horses were purified as described in [33]. The hyperimmue sera were adjusted to pH 3.5. Acidified equine antisera were digested with pepsin (porcine, Sigma) solution (50 mg/mL pepsin in distilled water, stored frozen at -20°C) at 37°C for 36 h. The digestion was terminated by titrating to pH 6.0 with the 50 mM piperazine base solution. Centrifuge at 2750 × g (4–10°C) to remove the precipitate. Tangential flow diafiltration was then performed to remove the bulk of low molecular weight digestion products. The digested antisera were washed with at least 15 volumes of diafiltration buffer/buffer A (20 mM piperazine, 150 mM NaCl, pH 6.0) on the tangential flow difiltration rig (VivaFlow50, Vivascience) with a 50 cm^2^, 30 000-Da nominal molecular weight cut-off membrane and concentrated to ~100 ml total volume. Diafiltrated digests were then passed through a column of Q Sepharose Fast Flow to remove the residual acidic aggregates and pepsin. All the unbound material, corresponding to the purified F(ab')_2_, was collected and stored at 4°C. Then eluted fractions (peak I) were concentrated with the same tangential flow diafiltration equipment and to obtain the final product with the desired concentration. The final F(ab')_2 _products were dissolved in PBS (pH 7.0, supplemented with 0.007% mercurothiolate), and their protein concentration and purity were determined by BCA method and folium scan, respectively.

### SDS-PAGE

Non-reducing SDS-PAGE gels using the Laemmli buffer system (1970) [34] were performed to check for traces of undigested IgG or large partially digested albumin fragments.

### ELISA

Total purified F(ab')_2 _was measured by an indirect enzyme-linked immunosorbent assay (ELISA) using whole purified H5N1 as coating antigen in a tetramethylbenzidine (TMB) system. Microwell plates were coated overnight at 4°C with each of the purified influenza H5N1 virus at 1 *μ*g/mL in carbonate-bicarbonate buffer (pH 9.6). The wells were washed three times with 0.05% Tween 20 in PBS (PBS-T) and then blocked with 5% non-fat milk in PBS-T at 37°C for 1 h. Following three washes with PBS-T, serum samples diluted were added and incubated at 37°C for 1 h. Following five washes, HRP-conjugated goat anti-horse IgG (Sigma) diluted 2000-fold in PBS-T was added to detect the bound antibodies. Following incubation at 37°C for 1 h, the plates were washed as above and the substrate tetramethylbenzidine (TMB) solution (Sigma) was added to the wells to generate the color. After incubation at room temperature for 30 min, the reaction was stopped by adding 2 mmol/L H_2_SO4. The absorbance value at 450 nm (A450) was determined with an ELISA reader (Model 550, BioRad, USA). Antibody titer was defined as the highest dilution of F(ab')_2 _at which the A450 ratio (A450 of negative serum) was greater than 2.0.

### HI test

F(ab')_2 _fragments were tested for antibodies to the influenza H5N1 virus Guangdong strain by hemagglutination-inhibition (HI) according to the operating procedures used in avian influenza virus recommended by World Health Organization (WHO) in 2002 [35]. HI was assessed using 25 *μ*l each of a series of F(ab')_2 _dilutions 1:2, and 25 *μ*l of HA antigen, standardized at 4 hemagglutination units (HAU) by hemagglutination titration, were added. The mixture was incubated for 1 h at room temperature, 50 *μ*l of 1% chicken erythrocytes were added and the plate was gently shaken. The HI titer was recorded after incubation for 1 h at room temperature and is expressed as the reciprocal of the F(ab')_2 _dilution that inhibited hemagglutination.

### Virus neutralization activity in vitro

Neutralizing antibody titer of F(ab')_2 _was determined by micro-cytopathic effect (CPE) neutralizing test with H5N1 virus Guangdong strain according to WHO protocols [35]. The F(ab')_2 _fragments against H5N1 virus were diluted in two-fold serially from 1:10 to 1:5120. The antibody solutions (100 *μ*l) were mixed in 1:1 (v/v) with suspension containing 100 TCID_50 _of highly purified H5N1 virus (10^8.5 ^TCID_50_/ml) particles and incubated at 37°C for 1 h. The virus-antibody mix was then transferred onto MDCK cell monolayers in 96-well plates at 37°C for 1 h subsequently. Washed with MEM maintenance medium, each well was added by 100 *μ*l MEM maintenance medium, and then incubated at 37°C in 5% CO_2 _incubator. Positive and negative controls were set as 'virus control' (with 100 TCID_50 _H5N1 virus only), 'normal cells control' [without virus or F(ab')_2_] and 'normal horse antibody control'. CPE status was observed every 24 h for 5 days. The neutralizing antibody titer was expressed as the reciprocal of the highest F(ab')_2 _dilution which gave 50% neutralization of 100 TCID_50 _of virus. The experiment was repeated three times.

### Therapeutic activity in vivo

Female BALB/c mice, 6–8 weeks old (provided by the Animal Centre of Sun Yat-sen University, Guangdong, China), were housed within separate negative-pressure stainless steel isolators in a high-containment BSL-3 agriculture facility. Feed and water were provided ad libitum. Approval for animal experiments was obtained from the institutional animal welfare committee.

The mice were randomized to 4 groups (ten mice per group) and infected with 50 *μ*l of H5N1 virus (10^8.5 ^TCID_50_/ml) by intranasal route. Twenty-four hours later, 3 groups of mice were injected intraperitoneally with 50, 100, 200 *μ*g anti-H5N1 F(ab')_2 _fragments, respectively. A negative control group of mice received normal horse sera (200 *μ*g). The survival of mice following the lethal challenge was scored each day for 14 days.

## Results

### Preparation of F(ab')_2 _fragments

**Figure 1:**
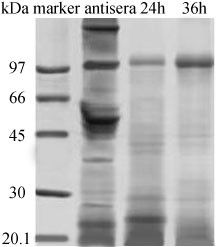
The digestion of equine antiserum with pepsin, as assessed by SDS-PAGE (10%) under non-reducing conditions. Digestion samples at corresponding time points, with molecular weight markers (first lane): 97 kDa, 66 kDa, 45 kDa, 30 kDa, 20.1 kDa, respectively.

**Figure 2:**
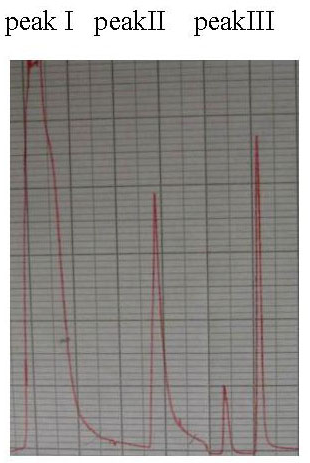
Removal of high molecular weight aggregate and pepsin by anion-exchange chromatography. Q-Sepharose FF ion-exchange separation of a diafiltrated pepsin digested antiserum. Peak I: F(ab')_2_, Peak II: high molecular weight aggregate and Peak III: pepsin.

The equine hyperimmune sera were digested with pepsin. SDS-PAGE showed that digestion within 36 h completely eliminated the high molecular weight material (e g. albumin and transferrin bands and the intact IgG), and only F(ab')_2 _band (~100 kDa) and very low molecular weight material was observed (Fig. 1). Following digestion, tangential flow diafiltration of the digested material was performed to remove all of the molecular weight lower than F(ab')_2_, leaving principally F(ab')_2 _and a small quantity of high molecular weight aggregate. Finally, the anion-exchange chromatography was then performed to remove the residual high molecular weight aggregate (acidic contaminants and pepsin). Diafiltered digests in diafiltration buffer (20 mM piperazine, 150 mM NaCl, pH 6.0) were separated into three peaks by anion exchange chromatography (Fig. 2). The first peak, which passed straight through the column, constituting ~90% of the material, containing the F(ab')_2 _fragments. Peak II, the acidic high molecular weight aggregate material was eluted by 200 ml buffer B. Peak III, the highly acidic pepsin was eluted by 20 ml buffer B. Material from the unbound peak (I) was then concentrated with a 30-kDa nMWCO ultrafilter and typically gave a final yield of 16 g F(ab')_2_/L antisera. The purity of F(ab')_2 _fragments reached over 90%, as measured by the folium scan method. The product obtained above was dissolved in a suitable volume of PBS to adjust the protein concentration to 2.0 mg/ml.

ELISA result showed that the specific activity of F(ab')_2 _fragments reached 1:5120 after pepsin digestion, ultra-filtration and anion-exchange chromatography.

### Protective efficacy of anti-H5N1 F(ab')_2 _in vitro

**Figure 3:**
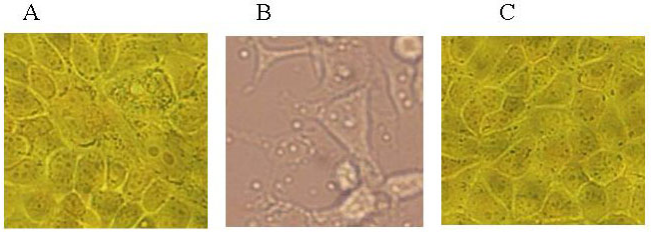
Photographs of micro-cytopathic effect neutralization tests. The F(ab')_2 _against H5N1 virus was diluted into two-fold serial dilutions, and incubated with an equal volume of active H5N1 virus dilution (100 TCID50). After neutralization, each mixture was added to MDCK cell monolayers in micro-plates, and incubated at 37°C to observe CPE status. These photographs showed the morphologic changes of MDCK cells at 72 h after infection. (A) Cell control (no CPE); (B) cell morphologic changes infected with the H5N1 virus; (C) MDCK cells protected from infection of H5N1 virus by anti-H5N1 F(ab')_2_.

The purified F(ab')_2 _was tested for HI activity against the lethal H5N1, and the HI antibody titer was determined as 1:1024. A virus neutralization assay was also included, infection of MDCK monolayer cells was carried out as described in Materials and methods. Fig. 3 displayed the neutralization photographs at 72 h with the F(ab')_2_. Compared with cell control (Fig. 3A), under the neutralization of 1:2048 dilution, the cells presented morphologic changes with about 50% CPE, which were calculated as the neutralization titres for F(ab')_2 _against the Guangdong H5N1 virus strain. CPE developed in virus controls (Fig. 3B), while anti-H5N1 F(ab')_2 _could protect MDCK cells from death of H5N1 virus infection and no CPE was observed (Fig. 3C).

### Effectiveness of passive immunotherapy with equine anti-H5N1 F(ab')_2 _administrated intraperitoneally

To verify the presumption that the prepared anti-H5N1 F(ab')_2 _fragments will have therapeutic efficacy in mammals, we tested the *in vivo *effectiveness of the F(ab')_2 _fragments in a BALB/c mouse model that had been proven to be vulnerable to infection with H5N1 virus by the intranasal route and replicated equally well in the lungs of mice without prior adaptation [36].

**Figure 4:**
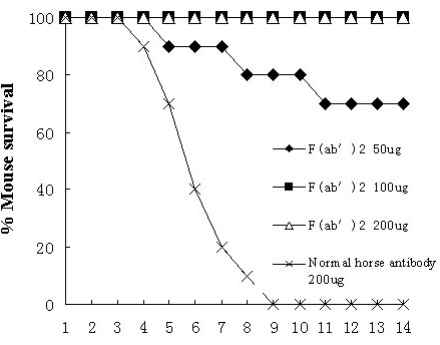
Efficacy of passive immunotherapy of influenza H5N1 virus infection by i.p. injection of F(ab')_2 _at dose of 50, 100 and 200 *μ*g/mouse at 24 h after intranasal challenge with the influenza Guangdong H5N1 virus strain.

We assayed the therapeutic efficacy of F(ab')_2 _fragments against the lethal dose of H5N1 viruses by intraperitoneal injection of 50, 100, 200 *μ*g F(ab')_2 _fragments/mouse using normal horse antibody as a control, 24 h after infection (Fig. 4). 50 *μ*g of anti-H5N1 F(ab')_2 _were required to give 70% protection. 100 and 200 *μ*g of anti-H5N1 F(ab')_2 _were required to give 100% protection. In contrast, the antibody-negative control (200 *μ*g of non-immune equine antibody) could not provide protection and the mice in this group died completely.

## Discussion

Over the past several years, cases of human infection with highly pathogenic H5N1 virus have raised international concern that we might face a global influenza pandemic in the near future. How can we arm ourselves against this pandemic threat? Although various kinds of vaccines against H5N1 virus are under development, there is still a long way to go from bench to bedside. As the latent phase of H5N1 virus infection is short, and the symptoms are hard to distinguish from those of the common cold, any delay in diagnosis and treatment could fatally jeopardize the patient's life. Once an individual is infected, administration of vaccine may be too late to elicit protective immunity. Meanwhile, we should seek multiple, mutually supportive intervention strategies to expand our weaponry against highly pathogenic H5N1 virus. Thus, it is imperative to develop a human H5N1 infection antidote that can provide immediate protection in such cases. In viral disease, antibodies obtained passively can deliver instant and short-term protection against infection regardless of the immune status of the host [37,38]. Development of human antibody against H5N1 virus is theoretically the ideal strategy to treat infection. However, it is difficult to obtain immune human donors. The heterogenous antibodies, for example, equine IgGs, have an advantage in this respect. Furthermore, one theoretically potential advantage of the polyclonal IgGs is the broader antigenic coverage and the lower likelihood of emergence of escape mutants. What's more, the heterogenous antisera are relatively economic and readily available upon request.

In this study, we reported the preparation of equine H5N1-specific F(ab')_2 _fragments and we observed their protective effects against highly pathogenic H5N1 virus infection in cultured mammalian cells. The *in vitro *neutralization assay showed that H5N1-specific F(ab')_2 _had protective effects on MDCK cells against H5N1 infection. A novel antidote has to be tested *in vivo *before entering clinical application. Accordingly, we evaluated the protective efficiency of equine H5N1-specific F(ab')_2 _against the H5N1 virus infection in a BALB/c mouse model. The results showed that 100 *μ*g of the F(ab')_2 _could protect 100% of mice infected with lethal challenge of H5N1 virus, if administrated 24 h after infection. Although the dose of F(ab')_2 _used here was relatively high compared with practical clinical application, this study may provide experimental data for preclinical studies regarding the effect of adoptive transfer of antibodies.

Nevertheless, the heterogeneous antibody possibly evokes a strong host immune response and inhibits its application in a clinical setting. The heterology of specific IgGs can be decreased through the preparation of F(ab')_2 _fragments by cutting off the Fc fragment. In this study, equine anti-H5N1 hyperimmune sera were purified by using a protocol for 'enhanced pepsin digestion'. Equine antisera were firstly digested with pepsin to remove a small amount of high molecular weight material. Tangential-flow diafiltration was then used as a convenient and highly effective method to remove the bulk of the low molecular weight contaminants (e.g. albumin, albumin fragments, and transferrin). However, diafiltration is ineffective at removing pepsin. Pepsin will bind to an anion-exchange matrix in the presence of 150 mM NaCl at pH 6.0. For most F(ab')_2 _fragments they pass straight through the column at this salt concentration. Further more, other acidic residual fragments, including the residual high molecular weight aggregates, also bind to the column at this salt concentration and are removed. Anion exchange was therefore used as a final purification step to remove the remaining pepsin and high molecular weight aggregates. Final yields of 16 g F(ab')_2_/L equine anti-H5N1 sera with a purity of over 90% were obtained, which compares favorably with the value of 6–14 g F(ab')_2_/L equine plasma reported [39]. In addition, this simple, high yield protocol for processing serum to highly purified F(ab')_2 _avoids the need for an initial or any subsequent salt precipitation step and can be utilised for either bench or large scale production of F(ab')_2 _notably for immunotherapeutic use.

Until we have an efficacious vaccine, specific anti-H5N1 agents, and effective epidemiologic control measures for H5N1 virus infection, highly pathogenic H5N1 virus is likely to be a major health threat to the world. In this article, we have attempted to provide an alternative pathway of prevention and treatment of H5N1 infection, and in doing so we hope that F(ab')_2 _purified from equine antiserum can play a potent role in combating the H5N1 virus. H5N1-specific F(ab')_2 _is polyclonal and polyvalent, so it may contain a wide variety of antibodies to variable or stable influenza H5N1 virus antigens, and may thus be of value for use in passive immunotherapy for prophylaxis and early treatment of influenza H5N1 infection. Influenza A H5N1-specific F(ab')_2 _fragments may potentially be used for the early treatment of avian influenza patients to reduce the severity of illness and the likelihood of H5N1 transmission to others.

## Competing interests

The author(s) declare that they have no competing interests.

## Authors' contributions

Jiahai Lu, Zhongmin Guo and Xinghua Pan conceived the study, planned the overall experimental design and wrote the manuscript. Guoling Wang, Dingmei Zhang, Liping Ouyang and Bingyan Tan carried out the experiments. Yanbin Li and Xinbing Yu advised in experimental design. All authors critically reviewed the manuscript.
